# Meat quality in broiler chickens fed on cowpea (*Vigna unguiculata* [L.] Walp) seeds

**DOI:** 10.1038/s41598-022-13611-5

**Published:** 2022-06-11

**Authors:** Georgeta Ciurescu, Lavinia Idriceanu, Anca Gheorghe, Mariana Ropotă, Reta Drăghici

**Affiliations:** 1 National Research & Development Institute for Biology and Animal Nutrition, Calea Bucureşti No. 1, 077015 Baloteşti, Ilfov Romania; 2 Research-Development Station for Plant Culture on Sands, 207170 Dăbuleni, Dolj Romania

**Keywords:** Nutrition, Lipids, Proteins

## Abstract

The study aimed to evaluate the effects of a diet containing untreated cowpea (CWP; Aura 26 variety) seeds as a protein source on quality parameters of chickens’ breast (PM; *Pectoralis major*) and thigh muscles (BF; *Biceps femoris*). A total of 240 Ross 308 broiler chickens were randomly allotted to two groups: a control group fed with soybean meal (SBM) and an experimental group fed with CWP included at 200 g/kg as a replacement of SBM. Each group consisted of six pens as replicates, with 20 chicks per pen. At 6 weeks of age, twelve birds/group were slaughtered. Compared to SBM group, the group fed CWP had higher (P < 0.0001) lightness (L*) and redness (a*) values of PM and BF muscles, the latter had also higher yellowness (b*, P < 0.0001). The collagen and protein contents were significantly higher in CWP group in both PM and BF muscles, while fat was lower (P < 0.001) only in BF muscle. The use of CWP into broilers’ diets did not negatively impact the textural properties, i.e., hardness, adhesiveness, cohesiveness, springiness, gumminess, chewiness, and resilience of PM and BF, showing similar values in both groups. Also, PM and BF muscles of birds fed CWP had significantly higher (P < 0.05) levels of C:18:3n-3 and C:20:5n-3 compared with birds fed SBM. The n-6/n-3 PUFA ratio was significantly lower in CWP group (11.72 and 7.00) compared to SBM (13.47 and 12.63) for both PM and BF muscles. These results indicate that CWP can be considered a promising protein source for broiler chickens’ feed.

## Introduction

In recent years, poultry meat has become one of the most popular and favourite protein sources due to its low price and high nutritional value. However, today's consumers have become increasingly concerned about the quality and safety of meat. In this direction, researchers are making efforts to improve the nutritional quality of meat, with the least financial effort and without affecting performance, especially for polyunsaturated fatty acids (PUFA), which is beneficial to human health^1^. Soybean meal (SBM) is often the major dietary plant protein source in broiler diets, and other protein sources other than SBM are used occasionally at competitive prices. Therefore, it is necessary to find new, non-traditional, low-cost feedstuffs to decrease the overall cost of poultry production.

In the past few years, the research interest for cowpea (CWP; *Vigna unguiculata* [L.] Walp) has grown. It is a crop with reasonable high protein content (203–394 g kg^−1^) as reviewed by Gonçalves et al*.*^2^, and which differs with the variety (Vasconcelos et al*.*^3^). Additionally, CWP seeds are rich in nutrients and nutraceuticals such as dietary fibre, antioxidants, PUFA and polyphenols, and minerals and vitamins^4–8^. However, CWP seeds possess some undesirable properties common to other legumes, such as methionine and cysteine deficiency, as well as considerable contents of antinutritional factors like protease inhibitors and phytic acid tannins, among others^9,10^. Nevertheless, in our previous study^11^, we have shown that new CWP cultivars (Ofelia drought-tolerant cv.) were characterized by low content of antinutritional factors (i.e., trypsin inhibitor activity). The authors also concluded that CWP seeds are a good source of protein and can partially replace SBM in broiler chicks' diets; no adverse effect on broiler growth performance and carcass traits were found. Therefore, CWP seeds could be an alternative source to soy and bean crops, especially under drought conditions, and are locally grown in Romania^12,13^. Up to date, we could not find any research about the effect of raw CWP on the quality of broiler meat; therefore, this research was an opportunity to test these diets effects on fatty acids (FA) deposition in breast and tight muscle.

The tested hypothesis is: the use of cowpea seeds in diets for broiler chicken has an effect on the quality of meat and fatty acids profile in breast and thigh muscles.

The study aimed to compare the meat traits, including physicochemical and textural properties as well as fatty acids composition, in broiler chickens fed on diets containing untreated CWP seeds as an alternative to SBM.

## Results

There were no significant differences (P > 0.05) in the values of pH_24_ for the breast muscles (PM; *Pectoralis major*) obtained from birds in the experimental group, which was fed with CWP compared to the control group (SBM; Table 1). However, the values of colour parameters for PM, such as lightness (L*) and redness (a*) from birds fed CWP, were significantly different (P < 0.0001) than in the SBM group, while the yellowness (b*) tended the increase (P = 0.070). Specifically, muscles from CWP group were characterized by higher L*, a* and b* (CWP: 62.47, 13.35 and 13.61; SBM: 55.44, 10.87 and 12.27, respectively). The analysis of the chemical composition of PM also showed significant differences in the content of protein (P < 0.0001) as well as collagen (P < 0.0001). No differences (P > 0.05; Table 1) were found in PM's moisture, and the fat level of broilers fed the SBM and CWP diets.

**Table 1:** Physicochemical parameters of breast and thigh muscles from 6-week-old broiler chickens.

Parameters	Group^A^		SEM	P-value
	SBM	CWP		
**Breast**				
pH_24_	5.97^a^	5.99^a^	0.01	0.470
Lightness (L*)	55.44^b^	62.47^a^	0.72	0.0001
Redness (a*)	10.87^b^	13.35^a^	0.54	0.0001
Yellowness (b*)	12.27^a^	13.61^a^	0.37	0.070
Protein (%)	21.68^b^	22.42^a^	0.12	0.0001
Fat (%)	1.72^a^	1.41^a^	0.13	0.274
Moisture (%)	76.60^a^	76.17^a^	0.14	0.117
Collagen (%)	0.69^b^	0.81^a^	0.02	0.0001
**Thigh**				
pH_24_	6.22^a^	6.23^a^	0.01	0.946
Lightness (L*)	55.65^b^	61.45^a^	0.59	0.0001
Redness (a*)	11.40^b^	13.20^a^	0.24	0.0001
Yellowness (b*)	9.28^b^	12.20^a^	0.43	0.0001
Protein (%)	18.13^b^	18.47^a^	0.08	0.016
Fat (%)	5.78^a^	5.20^b^	0.10	0.001
Moisture (%)	76.09^a^	76.33^a^	0.10	0.238
Collagen (%)	1.02^b^	1.08^a^	0.01	0.0001

*SEM* standard error of the mean.

^A^Group SBM: feed based on soybean meal; group CWP: feed based on raw cowpea seeds (*Vigna unguiculata* [L.] Walp, Aura 26 cv.).

^a,b^Means in the same row without same superscript differ significantly (*P* < 0.05).

The thigh muscles (BF; *Biceps femoris*) obtained from birds fed with CWP were characterized by comparable pH_24_ after slaughter than the SBM group (Table 1). All color values of BF from birds fed CWP were significantly higher (P < 0.0001) compared to birds fed SBM. However, the lightness (L*) redness (a*) and the yellowness (b*) of BF differed depending on the source of protein in the feed. Moreover, dietary protein sources influenced the BF protein, fat, and collagen content (Table 1). BF muscles collected from broilers fed CWP diet registered a significantly higher (P = 0.016) content of protein as well as collagen (P < 0.0001), while the fat level was lower (P < 0.001) compared with birds fed SBM diet. There were no significant differences (P > 0.05) in the moisture content for the BF muscles between broilers fed SBM and CWP diets.

The effect of different protein sources on the texture profile analysis (TPA) of PM and BF is shown in Table 2. Overall, the dietary inclusion of CWP into broilers’ diets did not negatively impact the TPA attributes of PM and BF, which showed similar values in both groups. Hardness, adhesiveness, cohesiveness, springiness, gumminess, chewiness, and resilience values were not different (P > 0.05) among groups in PM and BF samples.

**Table 2:** Textural properties of breast and thigh muscle from 6-week-old broiler chickens.

Parameters	Group^A^		SEM	P-value
	SBM	CWP		
**Breast**				
Hardness (g)	2602.50^a^	2456.43^a^	176.47	0.701
Adhesiveness (mJ)	0.27^a^	0.30^a^	0.03	0.682
Cohesiveness	0.21^a^	0.25^a^	0.01	0.097
Springiness (mm)	2.20^a^	2.22^a^	0.06	0.917
Gumminess (g)	605.86^a^	705.43^a^	59.15	0.422
Chewiness (mJ)	13.11^a^	15.60^a^	1.52	0.435
Resilience	0.21^a^	0.20^a^	0.06	0.839
**Thigh**				
Hardness (g)	4190.83^a^	3508.33^a^	845.15	0.732
Adhesiveness (mJ)	0.37^a^	0.57^a^	0.17	0.612
Cohesiveness	0.34^a^	0.31^a^	0.08	0.873
Springiness (mm)	3.73^a^	2.45^a^	0.46	0.184
Gumminess (g)	1969.33^a^	1241.67^a^	613.08	0.611
Chewiness (mJ)	85.63^a^	29.37^a^	28.01	0.372
Resilience	0.21^a^	0.23^a^	0.02	0.519

*SEM* standard error of the mean.

^A^Group SBM: feed based on soybean meal; group CWP: feed based on raw cowpea seeds (*Vigna unguiculata* [L.] Walp, Aura 26 cv.).

^a,b^Means in the same row without same superscript differ significantly (*P* < 0.05).

Data in Tables 3 and 4 indicate that the use of different protein sources in the diet of broiler chickens can influence the FA composition in PM and BF samples. As expected, the predominant FA in the PM and BF of the broiler chickens fed both the SBM and CWP diets was C18:1n9 (PM: 34.89 vs 35.14% of total FA, respectively; BF: 38.87 vs 37.72%, respectively), followed by C16:0 (PM: 21.21 vs 22.30% of total FA, respectively; BF: 27.00 vs 25.12%, respectively) and C18:2n-6 (PM: 22.74 vs 22.38% total FA; BF: 15.07 vs 15.82% total FA). Alpha-linolenic acid (ALA, C18:3n-3) is the precursor of long-chain n-3 PUFA such as eicosapentaenoic acid (EPA, C20:5n-3), docosapentaenoic acid (DPA, C22:5n-3), and docosahexaenoic acid (DHA, C22:6n-3), which are commonly referred to as n-3 or omega-3 PUFA. In this study, PM from birds fed CWP diets were characterized by a significantly higher content of ALA (P = 0.005) and EPA (P = 0.016) compared to the SBM diet (Table 3). Moreover, the proportion of total n-3 PUFA was slightly higher in chickens fed CWP (2.20) than in chickens fed SBM (2.00), but the difference was not significant (P > 0.05). The n-6/n-3 PUFA ratio was significantly lower (P = 0.045) in breast muscles from CWP group (11.72) compared to SBM (13.47). There were no significant differences (P > 0.05) in the total SFA and MUFA contents for the PM muscles between broilers fed SBM and CWP diets, although feeding CWP resulted in a significantly higher (P < 0.01) content of C14:0 and C16:0, while the C18:0 was lower (P = 0.002; Table 3).

**Table 3:** Fatty acids composition (% of total fatty acids) in breast muscles from 6-week-old broiler chickens.

Fatty acid	Group^A^		SEM	P-value
	SBM	CWP		
Caprylic (C8:0)	0.03^a^	0.02^a^	0.01	0.374
Capric (C10:0)	0.05^a^	0.03^a^	0.01	0.145
Lauric (C12:0)	0.01^a^	0.04^a^	0.01	0.140
Myristic (C14:0)	0.53^b^	0.58^a^	0.01	0.003
Myristoleic (C14:1)	0.12^a^	0.12^a^	0.01	0.998
Pentadecanoic (C15:0)	0.54^a^	0.47^a^	0.02	0.110
Pentadecenoic (C15:1)	0.06^a^	0.09^a^	0.01	0.060
Palmitic (C16:0)	21.21^b^	22.30^a^	0.25	0.002
Palmitoleic (C16:1)	4.34^a^	4.40^a^	0.03	0.255
Heptadecanoic (C17:0)	0.12^a^	0.12^a^	0.01	0.795
Heptadecenoic (C17:1)	0.11^a^	0.10^a^	0.01	0.101
Stearic (C18:0)	7.44^a^	7.09^b^	0.10	0.002
Oleic (C18:1n-9)	34.89^a^	35.14^a^	0.22	0.641
Linoleic (C18:2n-6)	22.74^a^	22.38^a^	0.11	0.081
Arachidic (C20:0)	0.06^a^	0.01^a^	0.02	0.117
Gamma-linolenic (C18:3n-6)	0.27^a^	0.25^a^	0.01	0.158
Alpha-linolenic (C18:3n-3)	0.47^b^	0.61^a^	0.03	0.005
Linoleic conjugate (C18:2)	0.16^a^	0.07^a^	0.06	0.514
Octadecatetraenoic (C18:4n-3)	0.51^a^	0.53^a^	0.03	0.774
Eicosadienoic (C20:2n-6)	0.23^a^	0.20 ^a^	0.03	0.692
Eicosatrienoic (C20:3n-6)	0.58^a^	0.50^a^	0.04	0.366
Erucic (C22:1n-9)	0.05^a^	0.09^a^	0.03	0.510
Eicosadienoic (C20:2n-3)	0.61^a^	0.63^a^	0.01	0.559
Arachidonic (C20:4n-6)	2.55^a^	2.09^a^	0.14	0.108
Docosadienoic (C22:2n-6)	0.16^a^	0.11^a^	0.02	0.383
Docosatrienoic (C22:3n-6)	0.08^a^	0.09^a^	0.01	0.116
Eicosapentaenoic (C20:5n-3)	0.15^b^	0.18^a^	0.01	0.016
Lignoceric (C24:0)	0.16^b^	0.21^a^	0.01	0.006
Nervonic (C24:1n-9)	0.75^a^	0.64^a^	0.03	0.063
Docosatetraenoic (C22:4n-6)	0.20^a^	0.18^a^	0.01	0.184
Docosapentaenoic (C22:5n-3)	0.14^a^	0.14^a^	0.01	0.998
Docosahexaenoic (C22:6n-3)	0.10^a^	0.11^a^	0.01	0.643
Other	0.56^a^	0.46^a^	0.05	0.439
Total SFA^B^	30.14^a^	30.86^a^	0.11	0.064
Total MUFA^C^	40.32^a^	40.57^a^	0.23	0.613
Total n-6 PUFA^D^	26.81^a^	25.82^b^	0.26	0.033
Total n-3 PUFA^E^	2.00^a^	2.20^a^	0.06	0.142
n-6/n-3 PUFA ratio	13.47^a^	11.72^b^	0.47	0.045

*SEM* standard error of the mean.

^A^Group SBM: feed based on soybean meal; group CWP: feed based on raw cowpea seeds (*Vigna unguiculata* [L.] Walp, Aura 26 cv.).

^B^Total SFA: saturated fatty acids, sum of C8:0 + C10:0 + C12:0 + C14:0 + C15:0 + C16:0 + C17:0 + C18:0 + C20:0 + C24:0.

^C^Total MUFA: monounsaturated fatty acids, sum of C14:1 + C15:1 + C16:1 + C17:1 + C18: 1n-9 + C22:1n-9 + C24: 1n-9.

^D^Total n-6 PUFA: sum of C18:2n-6 + C18:3n-6 + C20:2n-6 + C20:3n-6 + C20:4n-6 + C22:2n-6 + C22:3n-6 + C22:4n-6.

^E^Total n-3 PUFA: sum of C18:3n-3 + C18:4n-3 + C20:2n-3 + C20:5n-3 + C22:5n-3 + C22:6n-3.

^a,b^Means in the same row without same superscript differ significantly (*P* < 0.05).

**Table 4:** Fatty acids composition (% of total fatty acids) in thigh muscles from 6-week-old broiler chickens.

Fatty acid	Group^A^		SEM	P-value
	SBM	CWP		
Caprylic (C8:0)	0.07^b^	0.12^a^	0.01	0.042
Capric (C10:0)	0.04^a^	0.10^a^	0.02	0.176
Lauric (C12:0)	0.04^a^	0.13^a^	0.03	0.121
Myristic (C14:0)	0.71^b^	0.90^a^	0.05	0.041
Myristoleic (C14:1)	0.15^a^	0.18^a^	0.01	0.161
Pentadecanoic (C15:0)	0.35^a^	0.43^a^	0.02	0.064
Pentadecenoic (C15:1)	0.18^b^	0.67^a^	0.12	0.015
Palmitic (C16:0)	27.00^a^	25.12^b^	0.44	0.005
Palmitoleic (C16:1)	6.23^a^	5.36^b^	0.20	0.0001
Heptadecanoic (C17:0)	0.08^b^	0.15^a^	0.02	0.024
Heptadecenoic (C17:1)	0.19^a^	0.17^a^	0.05	0.901
Stearic (C18:0)	6.13^a^	6.66^a^	0.28	0.399
Oleic (C18:1n-9)	38.87^a^	37.72^a^	0.42	0.192
Linoleic (C18:2n-6)	15.07^b^	15.82^a^	0.18	0.009
Arachidic (C20:0)	0.02^a^	0.08^a^	0.02	0.203
Gamma-linolenic (C18:3n-6)	0.09^a^	0.11^a^	0.01	0.479
Alpha-linolenic (C18:3n-3)	0.17^b^	0.37^a^	0.05	0.018
Linoleic conjugate (C18:2)	0.19^b^	0.35^a^	0.04	0.044
Octadecatetraenoic (C18:4n-3)	0.67^b^	1.10^a^	0.10	0.001
Eicosadienoic (C20:2n-6)	0.79^a^	0.48^b^	0.07	0.0001
Eicosatrienoic (C20:3n-6)	0.14^b^	0.19^a^	0.01	0.006
Erucic (C22:1n-9)	0.01^a^	0.02^a^	0.01	0.132
Eicosadienoic (C20:2n-3)	0.12^b^	0.16^a^	0.01	0.008
Arachidonic (C20:4n-6)	0.50^a^	0.55^a^	0.02	0.233
Docosadienoic (C22:2n-6)	0.11^b^	0.24^a^	0.03	0.049
Docosatrienoic (C22:3n-6)	0.17^b^	0.22^a^	0.01	0.039
Eicosapentaenoic (C20:5n-3)	0.24^b^	0.41^a^	0.04	0.006
Lignoceric (C24:0)	0.23^b^	0.37^a^	0.03	0.014
Nervonic (C24:1n9)	0.12^a^	0.14^a^	0.01	0.158
Docosatetraenoic (C22:4n-6)	0.02^b^	0.08^a^	0.02	0.035
Docosapentaenoic (C22:5n-3)	0.04^a^	0.23^a^	0.07	0.154
Docosahexaenoic (C22:6n-3)	0.10^a^	0.31^a^	0.08	0.206
Other	0.83^a^	1.08^a^	0.06	0.225
Total SFA^B^	34.67^a^	34.05^a^	0.48	0.405
Total MUFA^C^	45.74^a^	44.27^a^	0.54	0.127
Total n-6 PUFA^D^	16.89^b^	17.69^a^	0.19	0.013
Total n-3 PUFA^E^	1.34^b^	2.58^a^	0.30	0.011
n-6/n-3 PUFA ratio	12.63^a^	7.00^b^	1.29	0.002

*SEM* standard error of the mean.

^A^Group SBM: feed based on soybean meal; group CWP: feed based on raw cowpea seeds (*Vigna unguiculata* [L.] Walp, Aura 26 cv.).

^B^Total SFA: saturated fatty acids, sum of C8:0 + C10:0 + C12:0 + C14:0 + C15:0 + C16:0 + C17:0 + C18:0 + C20:0 + C24:0.

^C^Total MUFA: monounsaturated fatty acids, sum of C14:1 + C15:1 + C16:1 + C17:1 + C18: 1n-9 + C22:1n-9 + C24: 1n-9.

^D^Total n-6 PUFA: sum of C18:2n-6 + C18:3n-6 + C20:2n-6 + C20:3n-6 + C20:4n-6 + C22:2n-6 + C22:3n-6 + C22:4n-6.

^E^Total n-3 PUFA: sum of C18:3n-3 + C18:4n-3 + C20:2n-3 + C20:5n-3 + C22:5n-3 + C22:6n-3.

^a,b^Means in the same row without same superscript differ significantly (*P* < 0.05).

Likewise, the use of different protein sources in the diet of broiler chickens also, influenced the FA compositions in BF samples (Table 4). The significantly higher (P = 0.018) content of ALA and C18:4n-3 (P < 0.0001) as well as EPA (P = 0.006) and total n-3 PUFA content (P = 0.011) was observed in chickens’ BF muscle fed CWP compared with the SBM diet. The analysis of BF samples also, revealed a significantly lower (P = 0.002) n-6 to n-3 PUFA ratio in broilers fed CWP diet (1.30) compared to SBM (7.00). Regarding the content of total SFA and MUFA in BF muscles, they followed the same trend as those in PM samples (P > 0.05).

## Discussion

For both groups, PM and BF, pH_24_ values were in the range of raw meat (5.5–6.2) and similar to other studies^14^ who reported close values for breast muscle. This indicates that there were no quality problems with the breast meat from each dietary treatment group.

Meat colour is an essential quality parameter since it is directly perceived by the consumer. In our study, the change of protein source in the diet significantly influenced the colour of breast and thigh muscle. This has been confirmed by Laudadio et al*.*^15^, who reported differences in the values of redness (a*) and the yellowness (b*) of breast and leg muscles, which were higher in chickens fed a diet with the inclusion of other legume plants (i.e., faba bean) compared to broiler fed SBM. The authors suggested that higher values of yellowness (b*) in leg muscles could be attributed to different FA contents and the SFA/ PUFA ratio in these muscles. Another studies^16,17^ on pea or lupin as a substitute for SBM into broilers' diets also reported that the yellowness (b*) of leg muscles was significantly higher, suggesting a lower level of lipids. Likewise, a trend towards higher lightness (L*) of leg muscles was also reported by Kuzniacka et al*.*^18^, who investigated the effect of faba bean (*Vicia faba* var. minor) based diet. Our study found significantly higher values of lightness (L*), redness (a*) and yellowness (b*) for PM and BF from chickens fed a diet based on CWP. Even if BF of CWP group showed a higher lightness, the pH value was in the range for normal meat.

In our study, the use of CWP as a substitute for SBM had a significant effect on most of the chemical parameters (the content of protein, fat and collagen) of breast and thigh muscles from 6-weeks-old broiler chickens. Muscles collected from broilers fed CWP diet registered higher protein and collagen contents. Consistently with our study, Laudadio et al*.*^15^ reported significantly higher content of collagen in breast and leg muscles from broilers fed a diet containing dehulled-micronized fava beans (310 g/kg). On the other hand, Dal Bosco et al*.*^19^ reported significantly lower fat content in leg muscles from organic slow-growing chickens fed a diet containing faba bean, which was also confirmed in our study. There is a lack of relative information regarding the texture properties of CWP, and indeed, of legume plants. To the best of our knowledge, this is the first study about the influence of the CWP diet on texture attributes of muscles. Therefore, more study is needed to determine the effects of these seeds on textural properties in broiler meat. The TPA breast meat traits observed in the present research were overall satisfactory and in line with values reported for another legume, i.e., chickpea^20^.

Manipulating broiler chickens’ diets by adding different protein sources resulted in significant changes in the FA composition of breast and thigh muscle. No research appears to have been reported on the effect of raw CWP seeds on meat FA composition in broiler chickens. The present study provides new insights into the use of CWP in the diet of broiler chickens. Therefore, this subject should be considered a new investigation. The use of CWP influenced markedly the breast and thigh muscle FA content. The highest total n-3 PUFA content was observed in the chickens' breast (2.20 vs 2.00) and thigh muscle (2.58 vs 1.34) fed CWP compared to SBM. This result was associated with a significant increase in most of the n-3 FA's in PM and BF samples, especially for those that may have potential benefits to human nutrition, i.e., ALA and EPA. Similar results were noted in other experiments where broiler chickens were fed diets with different protein sources such as faba bean^15^ and raw lentil seeds^21^ as replacements for SBM. The authors of the cited studies reported higher total content of n-3 PUFA in the breast and leg muscles from chickens fed with these legumes than SBM; they also observed a reduction in the n-6/n-3 ratio. Recently, Paszkiewicz et al*.*^22^ also found that the substitution of SBM by chickpea seeds can affect the FA proportions in the subcutaneous fat tissue of chickens. Desirable changes in the lipid profile of meat, including an increase in total n-3 PUFA content, and a decrease in the n-6/n-3 PUFA ratio, were also observed when SBM was replaced with camelina meal^23^ or oil^24^ and camelina oil or seeds^25^ in diets for broiler chickens. On the other hand, Sirri et al*.*^26^ reported that partial replacement of SBM with faba beans affected the proportions of some FA but had no effect on lipid fractions that are believed to be essential for human health. The reasons for the above contradiction could be different combinations of feed ingredients or level of inclusion used for formulating diets in each experiment, or even various genotypes of chickens.

On the basis of the obtained results, it can be concluded that the use of CWP as an alternative to SBM in the diet of broiler chickens had a favorable effect on meat quality. It is important because where CWP can be grown locally, low-input farming systems would benefit from using this ingredient for broiler chicken’s feed. Additionally, CWP can be a cheaper source of PUFA and an efficient method for modifying the FA profile of the meat in a way that is beneficial according to the dietetic recommendations for humans.

## Methods

The Animal Ethics Committee approved the study protocol of the National Research and Development Institute for Animal Biology and Nutrition (INCDBNA-IBNA) Baloteşti, Romania, under the EU Directive 2010/63/EU and Romanian Law on Animal Protection. The slaughter of birds was carried out following the applicable rules on handling animals at the time of slaughter, including humane treatment. Also, the methods used in the meat quality tests were carried out by the current and commonly used methodology described in the “Material and methods” section. The study was conducted in compliance with the ARRIVE guidelines. We confirm that all methods on chickens' were carried out in accordance with relevant guidelines and regulations.

The cowpea seed Aura 26 variety used in this study is part of the collection of genotypes from the Research-Development Station for Plant Culture on Sands, Dăbuleni, Romania, and the National Commission approved registration for horticultural plants, Decision No. 92841/2012.

### Birds and diets

The study was conducted on 240 one-day-old healthy broiler chickens (Ross 308) divided into two groups [a control group (SBM) feed a diet with soybean meal as a source of protein, and CWP group (experimental) feed a diet with untreated cowpea seeds], with similar initial weights (46.5 ± 0.23 g) into 6 replications with 20 birds each. The nutritional composition of CWP of the Aura 26 variety is described in Table 5. The feeding program was divided into two feeding phases: starter (days 1–24) and finisher (days 25–42). Diets for each feeding phase were formulated to be isocaloric, isonitrogenous, with similar total lysine, total sulphur amino acids (TSAA; Table 6), calcium and available phosphorous, and to meet or exceed breeder guidelines (Ross 308, Aviagen Ltd., Midlothian, UK). Diets were manufactured in mash form, without the inclusion of growth promoters or antibiotics. However, narasin as a coccidiostat (Monteban G100, Elanco GmbH) and phytase (Axtra PHY 5000 L, Danisco Animal Nutrition, Marlborough, UK) as exogenous enzymes were included in premixes of all two experimental diets. Feed and water were provided ad libitum.

**Table 5:** Nutritional composition of cowpea (*Vigna unguiculata* [L.] Walp, Aura 26 variety) seeds added to broiler diets.

Nutrients		Nutrients	
DM	910.0	Fatty acid, % of total FAME	
CP, g/kg DM	288.4	Lauric (C12:0)	0.25
EE, g/kg DM	11.6	Myristic (C14:0)	0.37
CF, g/kg DM	51.1	Pentadecanoic (C15:0)	0.24
Ash, g/kg DM	45.5	Palmitic (C16:0)	24.11
Ca, g/kg DM	23.3	Palmitoleic (C16:1)	0.12
P, g/kg DM	62.8	Heptadecaenoic (C17:0)	0.33
AME^a^, MJ/kg	12.8	Stearic (C18:0)	5.66
**Antinutrients**		Oleic (C18:1n-9)	9.47
TIA, TIU/mg	10.8	Linoleic (C18:2n-6)	34.53
UA, pH change	0.29	Alpha-linolenic (C18:3n-3)	21.85
**Amino acids (%)**		Octadecatetraenoic (C18:4n-3)	0.23
Arginine	1.78	Eicosadienoic (C20:2n-6)	0.0
Histidine	0.71	Eicosatrienoic (C20:3n-6)	0.0
Isoleucine	1.20	Arachidonic (C20:4n-6)	1.24
Leucine	1.84	Heneicosenoic (C21:0)	1.17
Lysine	1.86	Docosatetraenoic (C22:4n-6)	0.43
TSAA	0.68	Total n-6 PUFA^b^	36.20
Phenylalanine	1.39	Total n-3 PUFA^c^	22.08
Threonine	1.22	n-6/n-3 PUFA ratio	1.64
Valine	1.15		

*DM* dry matter, *EE* ether extract, *CF* crude fibre, *Ca* calcium, *P* phosphorus, *TIA* trypsin inhibitor activity, *TIU* trypsin international units, *UA* urease assay, *TSAA* total sulphur amino acids.

^a^AME, calculated value European Table of Energy Values for Poultry Feedstuffs (WPSA, 1989).

^b^Total n-6 PUFA, a sum of C18:2n-6; C20:2n-6.

^c^Total n-3 PUFA, sum of C18:3n-3; C22:6n-3.

**Table 6:** Ingredient and chemical composition of diets (as-fed basis).

Items	Starter (d 1–24)		Finisher (d 25–42)	
	SBM	CWP	SBM	CWP
**Ingredient (%)**				
Corn	57.98	45.71	64.09	51.13
Soybean meal	31.00	23.00	25.20	17.30
Corn gluten	4.00	4.00	3.50	3.50
Cowpea, cv. Aura 26	–	20.00	–	20.00
Soybean oil	2.00	2.30	2.60	3.57
Monocalcium phosphate	1.67	1.61	1.45	1.36
Calcium carbonate	1.45	1.52	1.27	1.32
Salt (NaCl)	0.28	0.28	0.28	0.28
l-Lysine HCl	0.29	0.21	0.28	0.18
dl-Methionine	0.25	0.29	0.25	0. 28
Choline-chloride (50%)	0.08	0.08	0.08	0.08
Vitamin–mineral mixture^a^	1.00	1.00	1.00	1.00
**Calculated composition**				
ME (MJ/kg)	12.70	12.69	13.23	13.24
CP	22.0	22.0	19.50	19.50
Lysine, total	1.34	1.34	1.15	1.15
Lysine, digestible	1.20	1.22	1.04	1.03
TSAA, total	0.98	0.98	0.90	0.90
TSAA, digestible	0.89	0.90	0.83	0.83
Ca	0.90	0.91	0.78	0.79
Available P	0.45	0.45	0.39	0.39
Crude fat	4.92	5.10	5.65	6.77
Crude fibre	2.80	3.34	2.62	3.16

*SBM* soybean meal, *CWP* cowpea, *ME* metabolizable energy, *TSAA* total sulphur amino acids.

^a^Supplied per kg diet: 12,000 IU vitamin A, 5000 IU vitamin D3, 75 mg vitamin E, 3 mg vitamin K_3,_ 3 mg vitamin B_1_, 8 mg vitamin B_2_, 5 mg vitamin B_6_, 0.016 mg vitamin B_12_, 13 mg pantothenic acid, 55 mg nicotinic acid, 2 mg folic acid, 0.2 mg biotin, 120 mg Mn, 100 mg Zn, 40 mg Fe, 16 mg Cu, 1.25 mg I and 0.3 mg Se, 70 mg Monteban G100, 0.2 g Axtra PHY 5000 L (1000 FTU).

Chickens were kept in pens on shavings litter in a temperature-controlled room with pens of identical size (1.75 × 1.55 m). Room temperature was maintained at 34 °C for the first 5 days and then gradually reduced according to standard management practices until a temperature of 22 °C by using thermostatically controlled heaters, fans, and adjustable sidewall inlets. Lighting was provided for 23 h/day from 1 to 7D, and from 8D, the light decreased by 1 h a day until 20 h, according to EU legislation (EU Council Directive 2007/43/EC). Broilers were vaccinated at the hatch for Marek's, Newcastle, and Infectious Bronchitis Disease.

### Slaughtering procedures and muscle sampling

At 42 days of age, twelve birds (two birds/pen) from each feeding group (chosen based on pen average final live weight) were individually identified and weighed. The birds were electrically stunned before slaughter, exsanguinated by neck cut, scalded, and eviscerated. Chickens live weight was registered before slaughtering. A total of twelve breasts and twelve thighs were collected on their right and left sides, individually vacuum-sealed and refrigerated (4 ± 1 °C). Meat quality parameters (pH_24_, and color) were assessed on the *Pectoralis major* muscle (PM) on the right breast and the *Biceps femoris* muscle (BF) on the right thigh, while the left breast and thigh meat were frozen at − 20 °C until further analysis (instrumental TPA, proximate chemical composition and FA composition).

### Meat quality traits

#### pH value and instrumental colour measurements

After 24 h cold storage at 4 ± 1 °C, the pH value was measured in triplicate using a Hanna portable pH-meter (model HI 99163, Hanna Instruments, Romania), fitted with a spear-type electrode (FC 099 stainless steel blade tip) and an automatic temperature compensation probe. The color of the muscles was determined using a portable colorimeter (model CR 410, Konica Minolta Inc., Osaka, Japan) calibrated with a white ceramic tile on D65 illuminate. The results were expressed in the CIE Lab color space^27^. The lightness component is represented by L*, which ranges between 0 to 100, redness by a* and yellowness by b*, both of which have a range of −120 to + 120. For instrumental color determination, three measurements were performed on a fat-free surface area in different locations of each muscle (PM, BF).

#### Texture profile analysis

The texture measurements of raw chicken muscle samples were analyzed individually by a double cycle compression using a texture analyzer (Model CT3 BROOKFIELD Engineering Laboratories, Inc. MA, USA). To texture analysis, each muscle (PM and BF) was cut into 3-cylinder shapes with a diameter of 20 mm and a height of 15 mm. For an increased accuracy of the parameters reading, there were avoided any large areas of fat. The texture analyzer was equipped with a 50 kg load cell, a cylinder probe of 76.2 × 10 mm to compress the samples and a fixture base table. The probe moved towards the sample at a constant speed of 2.0 mm s^−1^ (pre-test), 1.0 mm s^−1^ (test), and 2.0 mm s^−1^ (post-test). The data was collected using Texture Pro CT Software.

#### Proximate chemical composition

PM and BF samples were minced, homogenized, and divided into two parts. A portion was used to perform NIR (near-infrared reflectance) spectroscopy analysis. The remaining part was frozen and afterwards analyzed for total lipid extracts and FA composition. NIR data were acquired using a DA6200 meat analyzer (PerkinElmer, Inc. MA, USA), with transmission spectroscopy that uses diode array detectors in the wavelength range of 850 to 1050 nm. Raw minced and homogenized samples from the PM and BF muscles were loaded into a magnetically coupled plastic sample dish of 14 mm height and a volume of 170 mL and analyzed for moisture, protein, fat and collagen contents. To minimize sampling error, we set two duplicates, and each replication was measured twice. Before the sample measurements, a polystyrene check sample was used to verify the optical performance. The averaged spectrum was then used in subsequent analysis.

#### Fatty acid composition

The FA content was assessed via fatty acid methyl ester (FAME) gas chromatography. The method used for FA composition were done in the same way as previously described by Ciurescu et al*.*^28^. In brief, FA from the total lipid extracts was converted to methyl esters by transesterification (in methanol containing 3% concentrated H_2_SO_4_, for 4 h at 80 °C). Methyl esters of FA were evaluated in a Perkin Elmer-Clarus 500 chromatograph equipped with a flame ionization detector (FID) fitted with a BPX70 capillary column (60 m × 0.25 mm × 0.25 µm film thickness). The column temperature was programmed at 5 °C/min from 180 to 220 °C. The carrier gas was hydrogen (35 cm/s linear velocities at 180 °C), and the splitting ratio was 1:100. The injector and detector temperatures used were 250 °C and 260 °C, respectively. Peaks were identified by injecting pure FAME standards; quantification was assessed using tridecanoic acid (C13:0) as an internal standard. The results were expressed as the percentage of the total detected FA. The ratio of n-6 PUFA to n-3 PUFA (n-6/n-3 PUFA ratio) was calculated.

### Statistical analysis

The data were processed with SPSS Statistics software, v.20.0 for Windows (IBM SPSS Statistics, Armonk, NY, USA). A one-way analysis of variance (ANOVA) was used. Results are reported as means and standard error of the mean (SEM). The effect of the diet on the meat quality parameters was analyzed using Student's *t*-tests for independent samples. Significance was declared at *P* < 0.05. A statistical trend was considered for 0.05 < *P* ≤ 0.10.

### Ethics

The Animal Ethics Committee approved the bird's care and used protocol at the National Research-Development Institute for Biology and Animal Nutrition (INCDBNA-IBNA), Balotești, Romania, following the principles of EU Directive 2010/63/EU as transposed to Romanian legislation on Animal Protection used for Scientific Purposes (Law no. 199/2018). Also, the methods used in the meat quality tests were carried out in accordance with the current and commonly used methodology described in the “Material and methods” section.
